# 1809. Seroprevalence of Tick-borne Diseases including Alpha-gal Allergy and Associations with Musculoskelatal Symptoms in a Population-based Cohort in Central North Carolina

**DOI:** 10.1093/ofid/ofad500.1638

**Published:** 2023-11-27

**Authors:** Diana Zychowski, Carolina Alvarez, Haley Abernathy Rodgers, Dana Giandomenico, Ross M Boyce, Amanda E Nelson, Scott Commins

**Affiliations:** University of North Carolina at Chapel Hill, Durham, North Carolina; University of North Carolina at Chapel Hill, Durham, North Carolina; University of North Carolina at Chapel Hill, Durham, North Carolina; University of North Carolina at Chapel Hill, Durham, North Carolina; University of North Carolina at Chapel Hill, Durham, North Carolina; University of North Carolina at Chapel Hill, Durham, North Carolina; University of North Carolina at Chapel Hill, Durham, North Carolina

## Abstract

**Background:**

The incidence of ehrlichiosis, spotted fever rickettsioses (SFR), and Alpha-gal syndrome (AGS) is high in North Carolina (NC). Post-infectious sequelae secondary to ehrlichiosis or SFR are uncommon, however patients often attribute persistent musculoskeletal symptoms, including arthralgia, to prior tick-borne exposure. It is uncertain if these symptoms represent long-term complications from SFR or ehrlichiosis, or non-anaphylactic AGS via the development of IgE to galactose alpha-1,3-galactose (alpha-gal IgE). Utilizing a population-based cohort, we sought to examine the potential association between prior exposure to *Ehrlichia*, *Rickettsia* and alpha-gal, and chronic musculoskeletal symptoms.

**Methods:**

A total of 488 Individuals from the 2017–2018 Johnston County Osteoarthritis study – a longitudinal, probability-based cohort – completed questionnaires regarding health status and environmental exposures, underwent physical assessment, and had serological testing for *Rickettsia*, *Ehrlichia chaffeensis*, and alpha-gal IgE. Multivariable models were used to estimate associations of interest for this cross-sectional study.

**Results:**

The overall weighted point prevalence for *Ehrlichia* IgG was 8.6% (95% CI: 5.9–11.3%), *Rickettsia* IgG was 17.1% (95% CI: 12.6–21.5), and alpha-gal IgE > 0.1 IU/mL was 19.6% (95% CI: 15.3–23.8) in Johnston County, NC. Only one individual had antibodies to *Rickettsia*, *Ehrlichia*, and alpha-gal IgE > 0.1 IU/mL. Forty percent of participants had detectable alpha-gal IgE > 0 IU/mL, while only 2.5% self-reported a diagnosis of alpha-gal allergy to red meat. Only alpha-gal IgE was associated with knee pain, aching and stiffness (PAS) (IDR 1.3, 95% CI: 1.09-1.56). Antibodies to *Rickettsia*, *Ehrlichia*, and alpha-gal were not associated with symptomatic radiographic knee osteoarthritis (OA).

**Figure:**
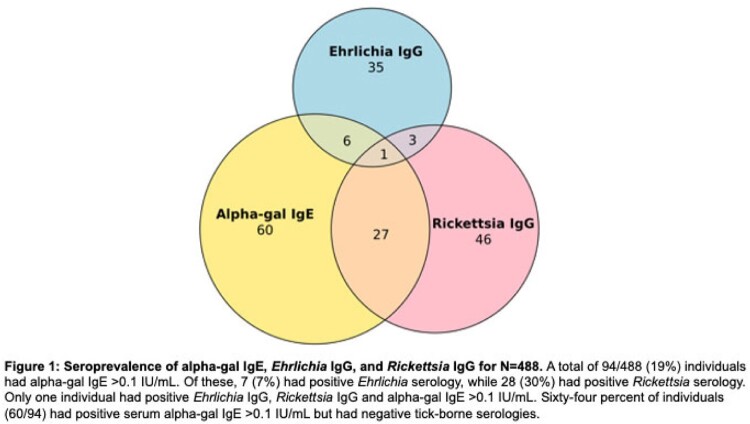

**Figure:**
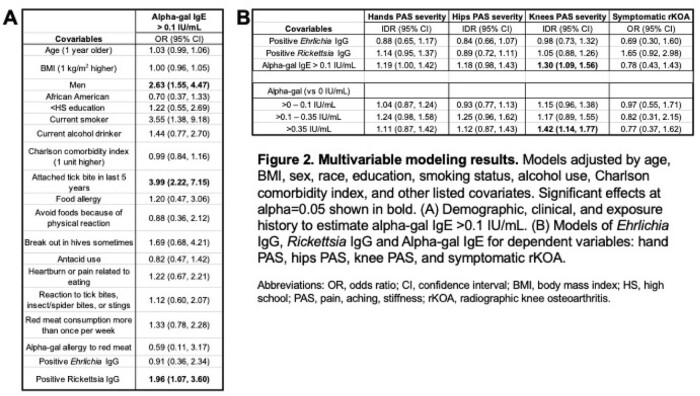

**Figure:**
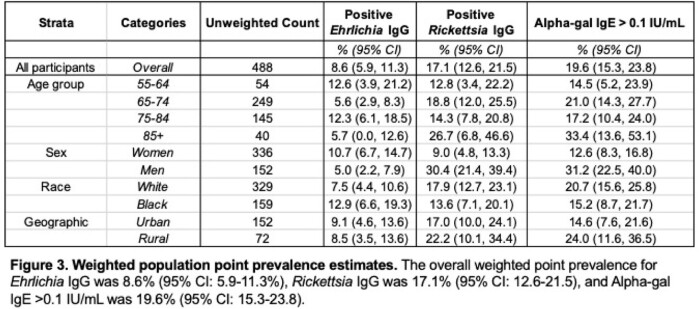

**Conclusion:**

Despite only 17% of individuals recalling a tick bite in the past 5 years, 52% had either i) alpha-gal IgE > 0 IU/mL; ii) positive *Rickettsia* IgG antibodies; and/or iii) positive *Ehrlichia* IgG antibodies, together, suggesting high levels of tick exposure. Positive *Ehrlichia* or *Rickettsia* tick-borne serologies did not predict OA or PAS within this population. Further investigation into extraintestinal manifestations of AGS is needed.

**Disclosures:**

**Scott Commins, MD, PhD**, Genentech: Advisor/Consultant|Revivicor: Grant/Research Support|Up to Date: Honoraria

